# Pharmacokinetic comparability of Prolastin^®^-C to Prolastin^® ^in alpha_1_-antitrypsin deficiency: a randomized study

**DOI:** 10.1186/1472-6904-10-13

**Published:** 2010-09-30

**Authors:** James M Stocks, Mark L Brantly, Laurene Wang-Smith, Michael A Campos, Kenneth R Chapman, Friedrich Kueppers, Robert A Sandhaus, Charlie Strange, Gerard Turino

**Affiliations:** 1 Department of Medicine, University of Texas Health Science Center at Tyler, 11937 US Hwy 271, Tyler, TX 75708-3154, USA; 2 University of Florida School of Medicine, Gainesville, FL, USA; 3 INDAPharma, LLC, Chapel Hill, NC, USA; 4 University of Miami School of Medicine, FL, USA; 5 University of Toronto, Toronto, Canada; 6 Temple University Hospital, Philadelphia, PA, USA; 7 National Jewish Health, Denver, CO, USA; 8 Medical University of South Carolina, Charleston, SC, USA; 9 St. Luke's-Roosevelt Hospital Center, New York, NY, USA

## Abstract

**Background:**

Alpha_1_-antitrypsin (AAT) deficiency is characterized by low blood levels of alpha_1_-proteinase inhibitor (alpha_1_-PI) and may lead to emphysema. Alpha_1_-PI protects pulmonary tissue from damage caused by the action of proteolytic enzymes. Augmentation therapy with Prolastin^® ^(Alpha_1_-Proteinase Inhibitor [Human]) to increase the levels of alpha_1_-PI has been used to treat individuals with AAT deficiency for over 20 years. Modifications to the Prolastin manufacturing process, incorporating additional purification and pathogen-reduction steps, have led to the development of an alpha_1_-PI product, designated Prolastin^®^-C (Alpha_1_-Proteinase inhibitor [Human]). The pharmacokinetic comparability of Prolastin-C to Prolastin was assessed in subjects with AAT deficiency.

**Methods:**

In total, 24 subjects were randomized to receive 60 mg/kg of functionally active Prolastin-C or Prolastin by weekly intravenous infusion for 8 weeks before crossover to the alternate treatment for another 8 weeks. Pharmacokinetic plasma samples were drawn over 7 days following last dose in the first treatment period and over 10 days following the last dose in the second period. The primary end point for pharmacokinetic comparability was area under the plasma concentration versus time curve over 7 days post dose (AUC_0-7 days_) of alpha_1_-PI determined by potency (functional activity) assay. The crossover phase was followed by an 8-week open-label treatment phase with Prolastin-C only.

**Results:**

Mean AUC_0-7 days _was 155.9 versus 152.4 mg*h/mL for Prolastin-C and Prolastin, respectively. The geometric least squares mean ratio of AUC_0-7 days _for Prolastin-C versus Prolastin had a point estimate of 1.03 and a 90% confidence interval of 0.97-1.09, demonstrating pharmacokinetic equivalence between the 2 products. Adverse events were similar for both treatments and occurred at a rate of 0.117 and 0.078 per infusion for Prolastin-C (double-blind treatment phase only) and Prolastin, respectively (p = 0.744). There were no treatment-emergent viral infections in any subject for human immunodeficiency virus, hepatitis B or C, or parvovirus B19 during the course of the study.

**Conclusion:**

Prolastin-C demonstrated pharmacokinetic equivalence and a comparable safety profile to Prolastin.

**Trial Registration:**

ClinicalTrials.gov Identifier: NCT00295061

## Background

Alpha_1_-antitrypsin (AAT) deficiency is an inherited autosomal disorder characterized by low blood levels of alpha_1_-proteinase inhibitor (alpha_1_-PI) and is one of the most common yet under recognized single-locus genetic diseases [1]. The disorder may lead to progressive severe emphysema that can manifest as early as the fourth decade of life [2,3].

Alpha_1_-PI protects the lung from damage by proteolytic enzymes (particularly neutrophil elastase) due to its action as an inhibitor of serine proteinases. Neutrophil elastase is released in the lung in response to inflammation and infection and can destroy the elastin in healthy pulmonary tissue. Neutrophil elastase is normally inactivated by alpha_1_-PI and the lung is protected, but in individuals with a deficiency of alpha_1_-PI, neutrophil elastase may cause irreversible destruction of pulmonary structures resulting in the development of emphysema.

The most specific approach to treating AAT deficiency is augmentation of low serum AAT levels with purified alpha_1_-PI through intravenous infusion in order to maintain plasma levels above 11 μM. Plasma alpha_1_-PI concentrations above a threshold of 11 μM are considered to be sufficiently protective against lung disease progression in individuals with AAT deficiency [4]. Prolastin^® ^(Alpha_1_-Proteinase Inhibitor [Human], Talecris Biotherapeutics, Research Triangle Park, NC, USA) is a plasma-derived protein that has been administered as augmentation therapy to individuals with AAT deficiency for more than 20 years. In 2003, two other preparations of alpha_1_-PI, Aralast (Baxter, Westlake Village, California, USA) and Zemaira (CSL Behring, Kankakee, Illinois, USA) were introduced in the United States.

Non-randomized observational studies have demonstrated a slower decline in lung function for subjects receiving Prolastin compared with those not receiving the augmentation therapy [5-7]. In addition, Prolastin has a proven tolerability [8-10] and pathogen safety record [11]. Scientific advances since the introduction of Prolastin have made it possible to incorporate modifications to the manufacturing process [12-14]. These include an additional cation exchange chromatography polishing step (to provide further purification), and improved ultrafiltration and diafiltration. Two new dedicated virus-reduction steps, solvent/detergent treatment and 15 nm nanofiltration, replace the pasteurization step in the Prolastin process.

These modifications to the Prolastin manufacturing process have led to the development of an alpha_1_-PI product, designated Prolastin^®^-C (Alpha_1_-Proteinase Inhibitor [Human]), with increased purity, a higher concentration of active alpha_1_-PI, and, as demonstrated in laboratory studies, a higher margin of safety from the risk of transmission of infectious pathogens.

The aim of this study was to investigate the pharmacokinetic characteristics of Prolastin-C in individuals with AAT deficiency and to compare these characteristics with those of Prolastin in support of licensing Prolastin-C. We report here the results of a multicenter, randomized, double-blind, crossover trial. We also assessed the tolerability and safety profile of Prolastin-C.

## Methods

### Subjects

Men and women aged ≥ 18 years were eligible for inclusion in the study if they had a documented diagnosis of congenital AAT deficiency with genotype PiZZ, PiZ Null, Pi Null Null, or other predefined "at-risk" alleles. Subjects must have received augmentation therapy with Prolastin for at least 1 month prior to study start. In addition, all subjects must have had documented alpha_1_-PI serum levels of < 11 μM prior to receiving any augmentation therapy and a forced expiratory volume in 1 second of 20% to 80% predicted value within the previous 6 months. All subjects provided written informed consent prior to study participation. Subjects were excluded from the study if they had a diagnosis of liver cirrhosis, any severe concomitant disease, history of anaphylaxis to plasma-derived alpha_1_-PI or other blood product, known immunoglobulin A (IgA) deficiency, or exacerbations of their pulmonary disease within 1 month of study entry. Use of systemic steroids prior to 2 weeks of receipt of study medication (excluding inhaled steroids used on a routine or as-needed basis) was also not permitted. The study was approved by the following local ethics review committees: University of Florida Health Science Center Institutional Review Board, National Jewish Medical Center and Research Center Institutional Review Board, University of Texas Health Center at Tyler Institutional Review Board, Western Institutional Review Board, and the Medical University of South Carolina Institutional Review Board for Human Research. The study was conducted in accordance with the Declaration of Helsinki and Good Clinical Practice guidelines.

### Study design

**Figure 1:**
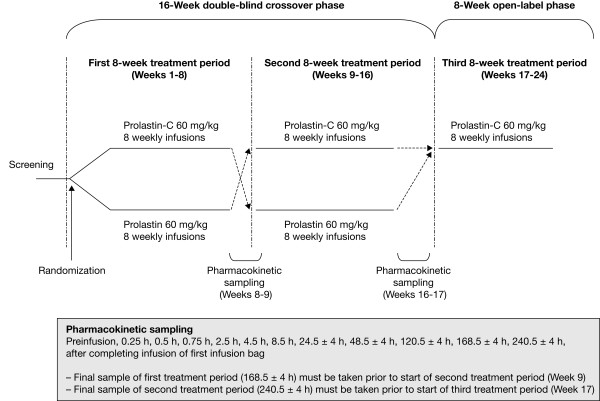
**Study design**.

This multicenter, randomized, double-blind study consisted of a 16-week crossover phase to evaluate the pharmacokinetic comparability between Prolastin-C and Prolastin (an initial 8-week treatment period and a second 8-week period based on the alternate treatment), followed by an 8-week open-label treatment phase with Prolastin-C only (Figure 1). Subjects were randomized in a 1:1 ratio. Each site was provided with randomization envelopes in numerical order and with the randomization number visible on the outside. Randomization envelopes were sent to the pharmacist with study medication. The 6-digit randomization numbers were assigned to subjects in ascending order at the baseline visit, when the subject's eligibility had been confirmed. Subjects received a weekly intravenous infusion of either 60 mg/kg body weight of functionally active Prolastin-C or 60 mg/kg functionally active Prolastin determined by potency assay during the first 8-week treatment period. Since Prolastin-C is reconstituted in half the volume of diluent compared with Prolastin, treatment was administered in 2 infusion bags of equal volume for each product: in the case of Prolastin-C, the first bag contained normal saline and the second Prolastin-C. For Prolastin total volume was split equally into 2 infusion bags. This was done in order to maintain the blinding. Prolastin-C was not split equally into 2 bags because the blinding methods would require dilution of the product to achieve volumes identical to Prolastin in each bag. The method used enabled assessment of the safety and tolerability of Prolastin-C as it would be used in clinical practice (ie, in a "real world" situation). Each infusion bag was administered over approximately 15 minutes such that the total infusion time for each treatment was 30 minutes.

### Blood sampling

In the first treatment period, a pre-infusion blood sample was drawn for pharmacokinetic analysis prior to the last dose of study medication (Week 8). Following this last dose, a total of 10 serial samples were drawn over 7 days at nominal time 0.25 h, 0.5 h, 0.75 h, 2.5 h, 4.5 h, 8.5 h, 24.5 h, 48.5 h, 120.5 h, and 168.5 h after completing infusion of the first infusion bag (Figure 1). Subjects were then crossed over to the alternate treatment. In the second treatment period, a pre-infusion blood sample was again taken prior to the last dose of study medication (Week 16), followed by 11 serial blood samples over 10 days after the last dose at nominal time 0.25 h, 0.5 h, 0.75 h, 2.5 h, 4.5 h, 8.5 h, 24.5 h, 48.5 h, 120.5 h, 168.5 h, and 240.5 h after completing infusion of the first infusion bag and before the start of the 8-week open-label treatment phase (Figure 1). In addition, blood samples were drawn for the determination of trough levels of alpha_1_-PI before the start of the infusion at Weeks 6, 7, and 8, and at Weeks 14, 15, and 16. During the open-label treatment phase (Weeks 17-24), all subjects received 60 mg/kg body weight Prolastin-C exclusively for 8 weeks.

Blood was collected into two tubes containing 4.5 ml of sodium citrate. The contents of the tubes were mixed gently and centrifuged at 2500 × g for 15 minutes. The plasma was transferred equally into two 3-ml cryovials using a disposable pipette, without disturbing the buffy coat (white blood cells) or the red blood cells. Each aliquot was required to contain an equal amount of sodium citrate solution and at least 1 ml of plasma. The samples were frozen immediately. The measurement of the alpha_1_-PI concentration in plasma was adjusted to account for the presence of sodium citrate.

### Pharmacokinetic parameters

For pharmacokinetic evaluation, plasma samples were analyzed for concentrations of alpha_1_-PI using (a) a potency (functional activity) assay to measure only the active concentration of protein capable of inhibiting neutrophil elastase (via a colorimetric method [15]), and (b) a content (antigenic) assay based on immunoprecipitation of soluble alpha_1_-PI antigen, using a corresponding alpha_1_-PI antiserum to measure both functionally active and inactive forms of the protein (by immunonephelometry [Behring Nephelometer BNII]). Both validated assays were applied to the analysis of each plasma sample. The concentration data are reported in the unit of mg/mL, and also in the unit of μM from the content assay in conformance with historical data. For the potency assay, the conversion factor is 1 mg/mL alpha_1_-PI equivalent to 19.2 μM. For the content assay, 1 mg/mL alpha_1_-PI is equivalent to 22.6 μM. The lower limit of quantification for alpha_1_-PI in plasma using the potency assay was 0.007 mg/mL and 0.07 mg/mL using the content assay.

Only the results of the potency assay were used for primary statistical analyses to determine pharmacokinetic comparability between the 2 treatments. The primary pharmacokinetic parameter used for treatment comparison was the area under the plasma alpha_1_-PI concentration versus time curve over 7 days' post-infusion (AUC_0-7 days_) at steady state (ie, after the last dose administered in each treatment period). Using the potency assay results for the primary pharmacokinetic parameter eliminates the inherent bias in plasma concentrations of total antigenic alpha_1_-PI between the 2 products with different antigenic content but administered at the same dose based on potency. Since the content assay has been used historically to report serum levels of alpha_1_-PI, especially trough levels, it was also performed to provide supportive data for historical comparison.

Other pharmacokinetic parameters determined for alpha_1_-PI included first observed peak plasma alpha_1_-PI concentration following drug infusion (C_max_), time to reach observed C_max _(t_max_), terminal half-life (t_1/2_), observed plasma alpha_1_-PI trough concentration prior to the start of infusions (C_trough_), and average trough concentration of alpha_1_-PI at steady state (mean C_trough_). The pharmacokinetic parameters of alpha_1_-PI at steady state were calculated at the end of the first and second 8-week double-blind treatment periods using noncompartmental pharmacokinetic methods with WinNonLin Professional Software, version 4.1 (Pharsight Corporation, Mountain View, California, USA).

### Safety profile

Safety data included frequency and nature of adverse events (AEs), exacerbations of pulmonary disease, vital signs, and laboratory data. Alpha_1_-PI antibody testing was also performed by Prolastin-C antibody screening enzyme-linked immunosorbent assay with a detection limit of 250 ng/mL. Subjects also underwent testing for human immunodeficiency virus (HIV), hepatitis B virus (HBV), hepatitis C virus (HCV), and parvovirus B19 using viral nucleic acid testing by polymerase chain reaction (PCR) at baseline and Weeks 9 and 17, and nucleic acid testing by PCR and viral serology testing at study end (Week 24). If a viral serology test result was positive at Week 24, or if any PCR result was positive at any time point during the study, then the sample retained at baseline was serologically tested.

### Statistical methods

The sample size of 20 subjects (10 subjects per treatment sequence) was sufficient to demonstrate comparability with regard to AUC_0-7days _with 90% power up to a standard deviation of 0.288 of the difference in the log_e _scale (this corresponds to a mean square error in the ANOVA ≤ 0.041) under the assumption of an expected mean treatment difference of 0 (or treatment ratio of 1), a lower equivalence limit of -0.223 (= log_e _0.8), an upper equivalence limit of 0.223 (= log_e _1.25), and a one-sided alpha of 0.05 (corresponds to a 90% confidence interval [CI]).

The study populations for analysis included the intent-to-treat population, which was defined as all randomized subjects; the safety population, which included all subjects who received any study medication; and the pharmacokinetic population, which comprised all subjects who received study medication and had sufficient plasma alpha_1_-PI concentration data to facilitate calculation of pharmacokinetic parameters.

Descriptive statistics were calculated for baseline variables and for each pharmacokinetic parameter determined from alpha_1_-PI concentration data by both potency and content assays. In order to compare the primary pharmacokinetic parameter (AUC_0-7 days_) between the 2 products, log_e_-transformed AUC_0-7 days _values were analyzed by analysis of variance, with treatment, treatment sequence, and study period as fixed effects, and subject (nested within sequence) as a random effect. A Wilcoxon Rank test was used to compare the AE rates between Prolastin-C and Prolastin. All statistical analyses and summaries were produced using SAS^® ^version 8.2 (or higher).

## Results

### Subject disposition and baseline characteristics

**Figure 2:**
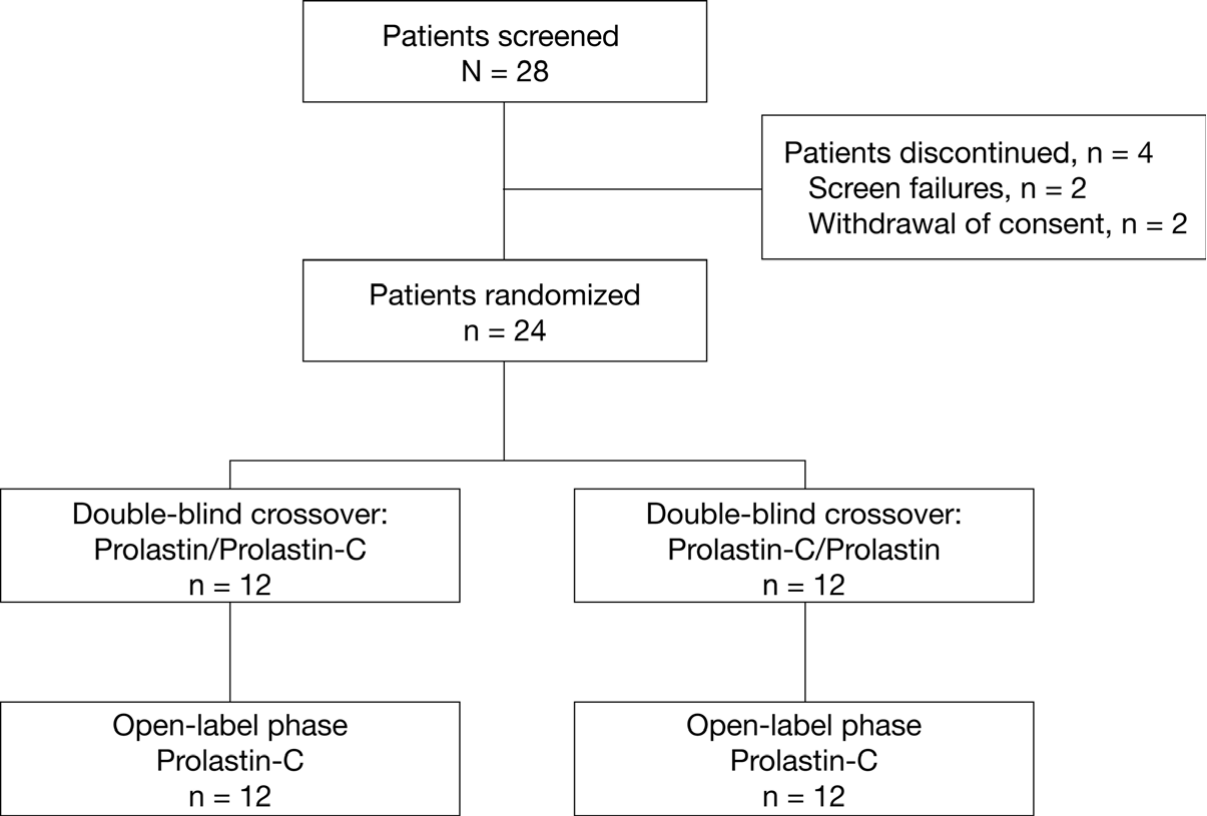
**Subject disposition**.

A total of 28 subjects were screened, 24 were randomized (12 to each treatment sequence, ie, Prolastin treatment followed by Prolastin-C treatment, or Prolastin-C treatment followed by Prolastin treatment), and 24 were eligible for pharmacokinetic analysis (Figure 2). Four subjects were discontinued prior to randomization (2 were screen failures and 2 withdrew consent). All randomized subjects completed both the double-blind treatment phase and the open-label phase. Subject demographics and baseline characteristics were similar between treatment sequences and are shown in Table 1. A diagnosis of severe AAT deficiency was confirmed by the presence of the PiZZ genotype in 23 of 24 subjects and by the genotype PiSZ in 1 subject. All subjects had received prior therapy with Prolastin.

**Table 1:** Subject Demographics by Treatment Sequence (ITT Population)

	Prolastin/Prolastin-C (n = 12)	Prolastin-C/Prolastin (n = 12)	All Subjects (n = 24)
Mean age, y (SD)	57.0 (9.33)	58.4 (6.86)	57.7 (8.04)

Mean body weight, kg (SD)	81.4 (15.37)	89.7 (19.49)	85.5 (17.67)

Male gender, n (%)	4 (33.3)	6 (50.0)	10 (41.7)

Female gender, n (%)	8 (66.7)	6 (50.0)	14 (58.3)

Time since AAT deficiency diagnosis, y (SD)	8.69 (6.96)	9.10 (6.02)	8.89 (6.37)

Mean pre-augmentation alpha_1_-PI plasma level, μM (SD)	5.29 (1.90)	4.92 (2.01)	5.11 (1.92)

Mean baseline alpha_1_-PI serum level, μM (SD)*	17.7 (3.26)	19.8 (4.38)	18.7 (3.91)

Mean FEV_1 _predicted, % (SD)	43.8 (13.2)	41.8 (13.8)	42.8 (13.3)

Deficiency genotype, n (%)			

PiZZ	12 (100)	11 (91.7)	23 (95.8)

PiSZ	0	1 (8.3)	1 (4.2)

Prior Prolastin therapy, n (%)	12 (100)	12 (100)	24 (100)

Medical history, n (%)^†^			

Obstructive pulmonary diseases^‡^			

COPD	8 (66.7)	7 (58.3)	15 (62.5)

Emphysema	6 (50.0)	7 (58.3)	13 (54.2)

Bronchiectasis	3 (25.0)	2 (16.7)	5 (20.8)

Asthma	3 (25.0)	3 (25.0)	6 (25.0)

Other conditions			

Headache	3 (25.0)	4 (33.3)	7 (29.2)

Pneumonia	2 (16.7)	3 (25.0)	5 (20.8)

Depression	2 (16.7)	3 (25.0)	5 (20.8)

Hypertension	2 (16.7)	3 (25.0)	5 (20.8)

COPD = chronic obstructive pulmonary disease; FEV_1 _= forced expiratory volume in 1 second; ITT = intent to treat. *By content (antigenic) assay (n = 22). ^†^Occurring in ≥ 5 subjects. ^‡^Pulmonary diagnosis reported in study documentation.

### Pharmacokinetic evaluation

Among the 24 subjects in the pharmacokinetic population, 1 subject missed the infusion at Week 8 when Prolastin-C was to be administered and 2 subjects receiving Prolastin at Week 8 did not have sufficient data points for determination of AUC_0-7 days _or other pharmacokinetic parameters due to sample hemolysis affecting the potency assay. Sample hemolysis affected the content assay of samples from 1 subject following Prolastin treatment.

#### Primary end point for pharmacokinetic comparability

**Figure 3:**
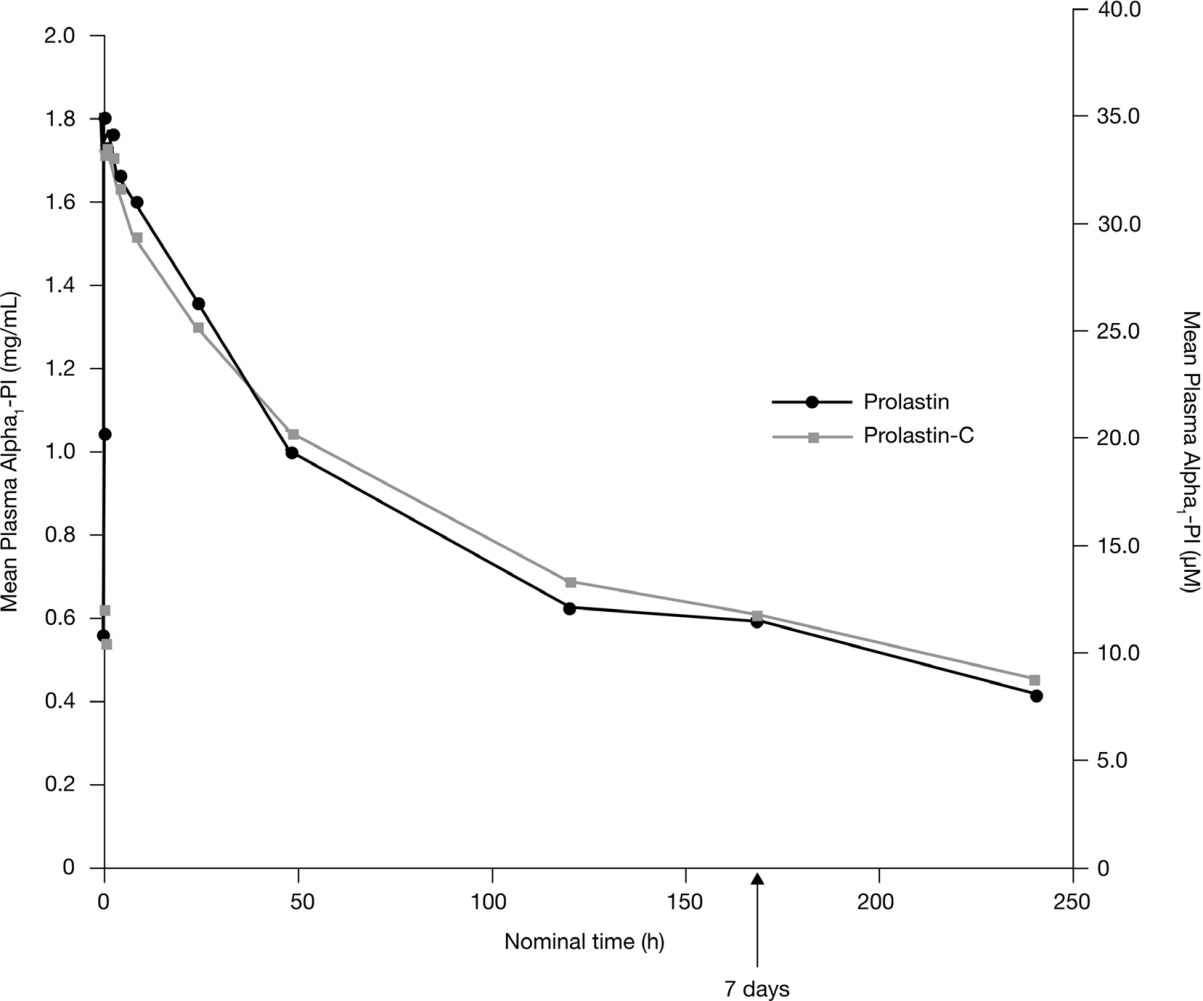
**Mean plasma alpha_1_-PI concentration versus time curves following treatment with Prolastin-C or Prolastin: potency [functional activity] assay results**.

The mean plasma alpha_1_-PI concentration versus time plots as determined by the potency assay show that plasma alpha_1_-PI concentrations versus time curves derived from the 2 products were almost superimposable (Figure 3). The mean AUC_0-7 days _was 155.9 mg*h/mL for Prolastin-C (n = 23) and 152.4 mg*h/mL for Prolastin (n = 22) (Table 2). The geometric least squares mean ratio of AUC_0-7 days _Prolastin-C versus Prolastin, had a point estimate and 90% CI of 1.03 and 0.97-1.09, respectively, for the potency assay results (Table 3). The 90% CI falls within the limit of 0.80-1.25, a criterion for concluding bioequivalence between treatments based on U.S. Food and Drug Administration (FDA) guidance [16].

**Table 2:** Summary of Pharmacokinetic Parameters of Plasma Alpha_1_-PI Determined by Potency [Functional Activity] Assay

	Prolastin-C 60 mg/kg	Prolastin 60 mg/kg
Mean AUC_0-7 days_, mg*h/mL (% CV)	155.9 (17)	152.4 (16)
	n = 23	n = 22

Mean C_max, _mg/mL (% CV)	1.797 (10)	1.848 (15)
	n = 22	n = 23

Median adjusted t_max_, hr (range)	0.673 (0.23-2.59)	0.820 (0.25-2.90)
	n = 22	n = 23

Mean t_½_, hr (% CV)	146.3 (16)	139.3 (18)
	n = 22	n = 22

CV = coefficient of variation.

**Table 3:** Ratio of Point Estimates and 90% CIs for AUC_0-7 days _for Prolastin-C versus Prolastin

	Geometric least squares mean ratio: Prolastin-C versus Prolastin			
	
	Potency assay		Content assay	
	
	Point estimate	90% CI	Point estimate	90% CI
AUC_0-7 days_, mg*h/mL	1.03	0.97-1.09	0.98	0.95-1.02

#### Other pharmacokinetic parameters

The other pharmacokinetic parameters were similar for both treatments based on the potency assay (Table 2). A slightly shorter median t_max _was measured for Prolastin-C compared with Prolastin (0.673 hour versus 0.820 hour, respectively). The 90% CI interval for the geometric least squares mean ratio of C_max _for Prolastin-C versus Prolastin based on the potency assay (0.92-1.04) was also within the limit of 0.80-1.25.

#### Pharmacokinetic parameters by content assay

**Figure 4:**
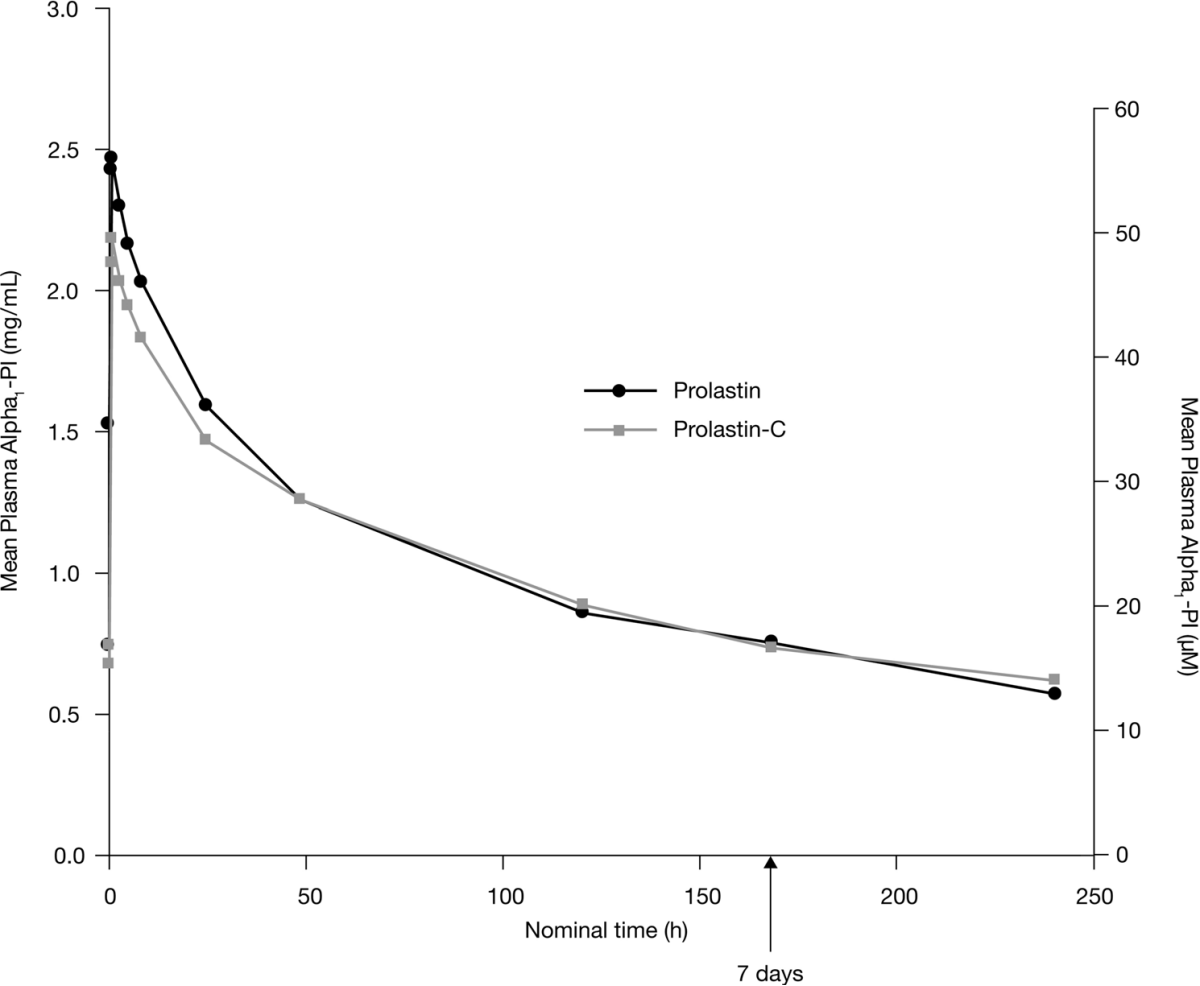
**Mean plasma alpha_1_-PI concentration versus time curves following treatment with Prolastin-C or Prolastin: content [antigenic] assay results**.

Using the content assay, the mean AUC_0-7 days _was 190.1 mg*h/mL for Prolastin-C (n = 23) and 194.8 mg*h/mL for Prolastin (n = 23), respectively. As an exploratory comparison, the 90% CI values for the geometric least squares mean ratio of AUC_0-7 days_, Prolastin-C versus Prolastin, also fell within the limit of 0.80-1.25 (Table 3). Mean plasma concentration versus time curves were similar between treatments with the exception of the distribution phase, where Prolastin elicited a higher C_max _compared with Prolastin-C (Figure 4). There were no major differences in other pharmacokinetic parameters for alpha_1_-PI measured between treatments based on the content assay except that median t_max _was slightly shorter for Prolastin-C (0.703 hour) than for Prolastin (0.955 hour). For comparison with historical data, C_trough _based on the content assay was evaluated (Table 4). All subjects, following each treatment, achieved mean C_trough _of plasma alpha_1_-PI above the historical target level of 11 μM.

**Table 4:** Summary of Steady-State Trough Concentrations of Plasma Alpha_1_-PI Determined by Content [Antigenic] Assay

	Prolastin-C 60 mg/kg	Prolastin 60 mg/kg
Mean C_trough_, μM (% CV)	16.9 (14)	16.7 (16)
	n = 23	n = 24

Subjects with mean C_trough _≥ 11 μM	23/23 (100%)	24/24 (100%)

One subject missed a Prolastin-C infusion at Week 8. Therefore, only 23 data sets were available for determination of C_trough _following Prolastin-C treatment.

### Safety results

#### Treatment exposure

A total of 188 weekly infusions of Prolastin-C and 192 weekly infusions of Prolastin were administered in the double-blind crossover phase. A further 192 weekly infusions of Prolastin-C were administered in the open-label treatment phase, resulting in a total of 380 weekly infusions of Prolastin-C throughout the whole study.

#### Adverse events

AEs occurring in the double-blind crossover and open-label phases are summarized in Table 5. During the entire study (double-blind crossover plus open-label phases), a total of 36 AEs were reported in subjects receiving Prolastin-C, giving a rate of 0.095 AEs per infusion. For the crossover phase, the rate of AEs per infusion was 0.117 in the subjects treated with Prolastin-C and 0.078 during Prolastin treatment (Table 5). A Wilcoxon Rank test gave a p-value of 0.744, which suggested no significant difference between the 2 treatments for the rates of AEs during the crossover phase. The most frequently reported AEs (occurring in ≥ 2 subjects) in the crossover phase were upper respiratory tract infection in subjects treated with Prolastin-C and headache and arthralgia in those treated with Prolastin. During the open-label phase, the most frequently occurring AEs in Prolastin-C-treated subjects were urinary tract infection and rales (Table 5). During Prolastin-C treatment, 2 AEs (2 episodes of pruritus in a single subject), representing a rate of 0.005 AEs per infusion, were considered to be related to study drug; 1 case occurred in the double-blind crossover phase and 1 in the open-label phase.

**Table 5:** Summary of AEs

	Double-blind Crossover Phase		Open-label Phase
	**Prolastin-C (n = 24)** **8 Weeks**	**Prolastin (n = 24)** **8 Weeks**	**Prolastin-C (n = 24)** **8 Weeks**

**AEs by subject**			

Any AE, n (%)	11 (45.8)	9 (37.5)	11 (45.8)

**AEs by total number and rate per infusion**			

Total number of AEs (rate per infusion)	22 (0.117)	15 (0.078)	14 (0.073)

Total number of AEs occurring in ≥ 2 subjects (rate per infusion)			

Upper respiratory tract infection	2 (0.011)	1 (0.005)	1 (0.005)

Urinary tract infection	1 (0.005)	0	2 (0.010)

Headache	1 (0.005)	2 (0.010)	0

Rales	0	0	2 (0.010)

Arthralgia	0	2 (0.010)	0

AEs leading to withdrawal	0	0	0

Total number of SAEs (rate per infusion)	0	2 (0.010)	0

Deaths	0	0	0

Total number of weekly infusions: Prolastin-C, 380 (double-blind plus open-label phases); 188 (double-blind phase only); 192 (open-label phase only). Prolastin, 192 (double-blind phase only).

The majority of AEs were considered to be mild to moderate in severity. Only 2 serious AEs were reported during the study: a single subject experienced severe spinal osteoarthritis and severe cervical spinal stenosis after 8 weeks of double-blind treatment with Prolastin, which were not considered to be related to study medication. No deaths or premature discontinuations due to AEs were reported in this study. Ten pulmonary exacerbations were observed in 8 patients during the study: 8 in the double-blind crossover phase (4 on Prolastin and 4 on Prolastin-C) and 2 in the open-label phase. Eight of the exacerbations were considered moderate (5 on Prolastin-C [3 in the crossover phase and 2 in the open-label phase] and 3 on Prolastin), and 2 were considered mild (1 each for Prolastin and Prolastin-C). No exacerbation of pulmonary disease was severe or considered to be a serious AE. All were resolved within approximately 2 weeks with drug therapy.

#### Viral testing

There were no treatment-emergent viral infections in any subject for HIV, HBV, HCV, or parvovirus B19 during the course of the study as measured by nucleic acid testing by PCR or viral serology. Evidence of a prior parvovirus B19 infection was observed in 18/24 subjects (75%). These subjects tested negative for parvovirus B19 IgM antibodies and positive for parvovirus B19 IgG antibodies at baseline and Week 24, and negative for parvovirus DNA throughout the study. In addition, these subjects did not exhibit any clinical symptoms of infection.

#### Other safety data

There were no clinically relevant differences between Prolastin-C and Prolastin with respect to laboratory assessments (hematology, clinical chemistry, and urinalysis parameters) or vital signs throughout the study. All study samples were negative for antibodies to alpha_1_-PI.

## Discussion

This study describes the first human administration of Prolastin-C, a new concentrated form of alpha_1_-PI prepared from human plasma for administration as augmentation therapy to individuals with AAT deficiency. Since it has increased purity and a higher concentration of alpha_1_-PI in comparison with Prolastin, Prolastin-C has the potential to be administered with a shorter infusion time and, therefore, may offer improved convenience for patients receiving weekly doses of alpha_1_-PI.

The study was designed to evaluate the pharmacokinetic profile of Prolastin-C and to assess the pharmacokinetic comparability of Prolastin-C to Prolastin in subjects with AAT deficiency. Bioequivalence was demonstrated between the 2 alpha_1_-PI preparations, both administered at a dose of 60 mg/kg body weight based on the potency assay. The 90% CI of the geometric least squares mean ratio for AUC_0-7 days _by potency assay, the primary end point, Prolastin-C versus Prolastin, was 0.97-1.09, which was within the limit that defines bioequivalence between 2 products according to FDA guidance, namely 0.80-1.25 [16]. This demonstration of bioequivalence between Prolastin and Prolastin-C provides clinicians with the confidence that Prolastin-C will have a comparable pharmacokinetic profile. Patients prescribed Prolastin-C will maintain similar overall plasma exposure to alpha_1_-PI as they had with Prolastin. In addition, Prolastin-C has improved purity and a higher concentration of alpha_1_-PI.

Augmentation therapy for AAT-deficient patients has been through intravenous administration of alpha_1_-PI with the dose amount based on the functionally active alpha_1_-PI in the drug product, measured by the functional activity assay. Historically, augmentation therapy has been evaluated by measuring the rise in serum concentrations of alpha_1_-PI using an antigenic content assay after administration of an alpha_1_-PI product. The antigenic content assay measures both functionally active and non-active forms of alpha_1_-PI. The functionally inactive forms may include oxidized, oligomerized, and polymerized forms of the alpha_1_-PI protein.

It has previously been shown that some alpha_1_-PI preparations have a higher antigenic content than functionally active content, which results in differences in the specific activity of alpha_1_-PI products. When dose is determined based on the amount of functionally active alpha_1_-PI, the product with a lower specific activity would be administered at a higher amount of total or antigenic content of alpha_1_-PI, potentially resulting in higher concentrations of the alpha_1_-PI antigenic content in plasma. Therefore, using the content assay to assess pharmacokinetic comparability between 2 products with different specific activities (eg, Prolastin with ≥ 0.35 mg functional alpha_1_-PI per milligram of total protein versus Prolastin-C with ≥ 0.8 mg per milligram of total protein) introduces an inherent bias in the comparison.

In order to eliminate this bias, the potency assay was employed to measure concentrations of functionally active alpha_1_-PI in the systemic circulation and to assess pharmacokinetic comparability between the 2 products (Prolastin-C and Prolastin), administered at the same dose amount of the functionally active alpha_1_-PI. Since the content assay has been used historically to measure systemic exposure to alpha_1_-PI for its protective effect to maintain plasma levels above a threshold of 11 μM [4], the results from the content assay, as well as those from the potency assay, were used in the graphical representation of plasma alpha_1_-PI concentration versus time following treatment with Prolastin-C or Prolastin (Figures 3 and 4). It should be noted that it is not possible, or appropriate, to compare results between 2 assay methods that measure different entities (ie, one for functionally active protein and the other for functionally active and inactive proteins), as different reference standards are used to establish the calibration curves.

While the primary end point for pharmacokinetic comparability of this study was based on data from the potency assay, results derived from the content assay also demonstrated pharmacokinetic comparability between the 2 alpha_1_-PI preparations, based on the 90% CI (0.95-1.02) of the geometric least squares mean ratio of AUC_0-7 days_, Prolastin-C versus Prolastin.

Mean values of other key pharmacokinetic parameters (including C_max_, t_max_, t_1/2_, and mean C_trough_) derived from the potency and content assays were also comparable between Prolastin-C and Prolastin. Analysis of the data for C_max _using the potency assay showed that the 90% CI values of the geometric least squares mean ratios for Prolastin-C versus Prolastin (0.92-1.04) were also within the limit defined by the FDA guidance for bioequivalence. This result is encouraging, even though the study was not designed to compare C_max _between products, because C_max _would vary with the rate of infusion, which is the case of this study (ie, 4 mg/kg/min for Prolastin-C and 2 mg/kg/min for Prolastin). Slightly higher C_max _of alpha_1_-PI concentrations were measured following Prolastin treatment compared with Prolastin-C treatment using the content assay. Prolastin was administered at a greater total protein "antigenic content" than Prolastin-C, which could potentially result in higher plasma concentrations of alpha_1_-PI antigenic content especially during the early phase shortly after infusion. The higher antigenic content of Prolastin results from its lower specific activity versus that for Prolastin-C.

A slightly shorter t_max _(by approximately 0.15-0.25 hour) was observed for Prolastin-C compared with Prolastin, using both the potency and content assays. This may be explained by the shorter infusion time for Prolastin-C (over 15 minutes) compared with Prolastin (over 30 minutes) because Prolastin-C was reconstituted in half the diluent of Prolastin, and therefore the infusion time for Prolastin-C was reduced by 50%. This slight difference in t_max _between the 2 treatments, mainly introduced by trial design, would not be clinically meaningful, considering how Prolastin-C will be used in the clinic. There were no substantial differences between Prolastin-C and Prolastin in mean t_1/2 _estimates for alpha_1_-PI based on the results of the potency assay (146.3 and 139.3 hours, respectively). The mean t_1/2 _values obtained using the content assay were also comparable between Prolastin-C and Prolastin. These values support the once-weekly dosing schedule for each treatment.

In this study, mean C_trough _concentrations for plasma alpha_1_-PI derived from the content assay were at levels that maintained a protective concentration of alpha_1_-PI. Furthermore, all subjects receiving either of the 2 treatments achieved mean C_trough _levels of alpha_1_-PI above the historical target threshold of 11 μM.

Both Prolastin-C and Prolastin were well tolerated, and there was no statistically significant difference between the rates of AEs per infusion between the 2 treatments. The safety profiles of Prolastin-C and Prolastin in this study were similar to those previously described for weekly Prolastin infusions. The observations with Prolastin-C are consistent with the known Prolastin risk profile, and no unexpected safety findings were reported. The results also confirmed the virus safety of Prolastin-C.

## Conclusion

For more than 20 years, Prolastin has been one of the mainstays of treatment for AAT deficiency. Compared with the Prolastin manufacturing process, the Prolastin-C modified process provides greater purity, a higher concentration of active alpha_1_-PI, and a high margin of safety from the risk of transmission of infectious pathogens. Given the pharmacokinetic comparability and similar safety profiles between Prolastin-C and Prolastin as demonstrated by this study, Prolastin-C is a safe and effective therapy for individuals with AAT deficiency.

## Competing interests

**Dr Stocks **has received grant support related to alpha-1 antitrypsin drug development from Baxter, CSL Behring, Kamada, and Talecris Biotherapeutics. He has been a consultant for Baxter, CSL Behring, and Talecris. He is on the speakers' bureau for Baxter and Talecris. **Dr Brantly **received grants in support of this research: National Heart, Lung and Blood Institute (NHLBI) K24 HL004456-5, University of Florida MO1 RR000082, and Talecris Biotherapeutics. **Dr Wang-Smith **is a pharmacokinetic consultant to Talecris Biotherapeutics, Inc, on a contract/consulting basis with fees. **Dr Campos **has received consulting fees from Talecris Biotherapeutics. **Dr Chapman **has received compensation in the past 3 years for consulting with AstraZeneca, Boehringer Ingelheim, CSL Behring, GlaxoSmithKline, Merck Frosst, Novartis, Nycomed, Pfizer, Roche, Schering Plough, and Talecris Biotherapeutics; has undertaken research funded by AstraZeneca, Boehringer Ingelheim, CSL Behring, Forest Labs, GlaxoSmithKline, Novartis, Parangenix, Roche, and Talecris; and has participated in continuing medical education activities sponsored in whole or in part by AstraZeneca, Boehringer Ingelheim, Grifols, GlaxoSmithKline, Merck Frosst, Novartis, Nycomed, Pfizer, and Talecris. **Dr Kueppers **has received grant support from CSL Behring, Baxter Healthcare, and Talecris Biotherapeutics. **Dr Sandhaus **has received grants from the Alpha-1 Foundation, the National Institutes of Health, and AlphaNet. He has been a consultant for Kamada, CSL Behring, Talecris Biotherapeutics, GTC Biotherapeutics, Dey Pharmaceuticals, and Arriva on AATD. **Dr Strange **has received grants from the National Institute of Health, Talecris Biotherapeutics, the Alpha-1 Foundation, and the Alpha-1 Association in the study of alpha-1 antitrypsin deficiency (AATD). He has been a consultant for Arriva, GTC Biotherapeutics, Talecris Biotherapeutics, and Gilead on AATD. He is on the speakers' bureau for Talecris Biotherapeutics and Baxter Healthcare. **Dr Turino **is a consultant to Talecris Biotherapeutics and has had research grant support from Boehringer Ingelheim Corp.

## Authors' contributions

JMS, FK, and RAS all contributed to study design and acquisition of data. They each participated in the analysis and interpretation of results and were involved in the preparation of the manuscript. MLB carried out the alpha1-PI assays, made substantial and significant contributions to study design, coordination, review of the data, and editing of the manuscript. LW-S made substantial and significant contributions to the conception, study design, protocol amendment, data analysis and interpretation of the pharmacokinetic data, identified critical issues over the bioanalytical methods and data discrepancies to be addressed, and wrote the Clinical Study Report for this study. In addition, she was instrumental in reviewing, commenting, drafting, and revising the manuscript. MAC contributed to the acquisition, analysis, and interpretation of data, and was involved in drafting of the manuscript and revising it critically for important intellectual content. KRC was involved in the analysis and interpretation of data and critically reviewed the manuscript. CS participated in study design and coordination and helped to edit the manuscript. GT participated in planning the study and reviewed the manuscript. All authors read and approved the final manuscript.

## Pre-publication history

The pre-publication history for this paper can be accessed here:

http://www.biomedcentral.com/1472-6904/10/13/prepub
